# Glia limitans superficialis oxidation and breakdown promote cortical cell death after repetitive head injury

**DOI:** 10.1172/jci.insight.149229

**Published:** 2021-10-08

**Authors:** Hannah D. Mason, Alexis M. Johnson, Nicole A. Mihelson, Panagiotis Mastorakos, Dorian B. McGavern

**Affiliations:** 1 Viral Immunology and Intravital Imaging Section and; 2 Department of Surgical Neurology, National Institute of Neurological Disorders and Stroke (NINDS), NIH, Bethesda, Maryland, USA.

**Keywords:** Immunology, Neuroscience, Neurological disorders

## Abstract

Repetitive mild traumatic brain injuries (mTBI) disrupt CNS barriers, the erosion of which has been linked to long-term neurodegenerative and psychiatric conditions. Although much attention has been devoted to CNS vasculature following mTBI, little is known about the glia limitans superficialis — a barrier of surface-associated astrocytes that helps protect the CNS parenchyma and maintain homeostasis. Here, we identify the glia limitans superficialis as a crucial barrier surface whose breakdown after acute repeat mTBI facilitates increased cell death and recruitment of peripheral myelomonocytic cells. Using intravital microscopy, we show that brain-resident microglia fortify this structure after a single mTBI, yet they fail to do so following secondary injury, which triggers massive recruitment of myelomonocytic cells from the periphery that contribute to further destruction of the glia limitans superficialis but not cortical cell death. We demonstrate, instead, that reactive oxygen species (ROS) generated in response to repetitive head injury are largely responsible for enhanced cortical cell death, and therapeutic administration of the antioxidant glutathione markedly reduces this cell death, preserves the glia limitans, and prevents myelomonocytic cells from entering the brain parenchyma. Collectively, our findings underscore the importance of preserving the glia limitans superficialis after brain injury and offer a therapeutic means to protect this structure and the underlying cortex.

## Introduction

Traumatic brain injuries (TBI) represent a dire global health and economic problem, impacting 69 million people worldwide and costing approximately $400 billion (USD) in associated medical fees annually (1, 2). Most of these injuries (~81%) are mild in nature and are not medically treated. Recent studies, however, have linked repetitive mild TBI (mTBI) sustained in sports and certain occupations to long-term neurodegenerative and psychiatric diseases (3–6). For example, in the United States, 110 out of 111 professional football players whose brains at autopsy were donated for research were found to have a neurodegenerative disease referred to as chronic traumatic encephalopathy (7).

Currently, no therapeutic has succeeded in improving outcomes after TBI (8). These failures likely stem from our incomplete understanding of injury pathophysiology. Physical barriers are crucial in protecting the brain from injury and disease, and TBI studies have focused on understanding the breakdown of specific barriers associated with meningeal and parenchymal vasculature (9, 10). Maintenance of vascular integrity in the CNS is important because its breakdown has been associated with disability following TBI (11). However, vasculature is not the only relevant barrier surface to consider. The glia limitans superficialis is a layer of surface-associated astrocytes that resides beneath the pia mater and serves as a barrier between cerebral spinal fluid space and CNS parenchyma (12). It covers the entire brain parenchyma, yet little is known about this important barrier surface in the context of TBI, despite the fact that it is permeabilized following brain injury (6, 13). We therefore set out to elucidate mechanisms of glia limitans superficialis breakdown after single and repetitive mTBI, with the hope of discovering a druggable pathway to preserve barrier integrity and mitigate adverse, postinjury outcomes.

## Results

### Repeat brain injury increases breakdown of the glia limitans superficialis.

**Figure 1:**
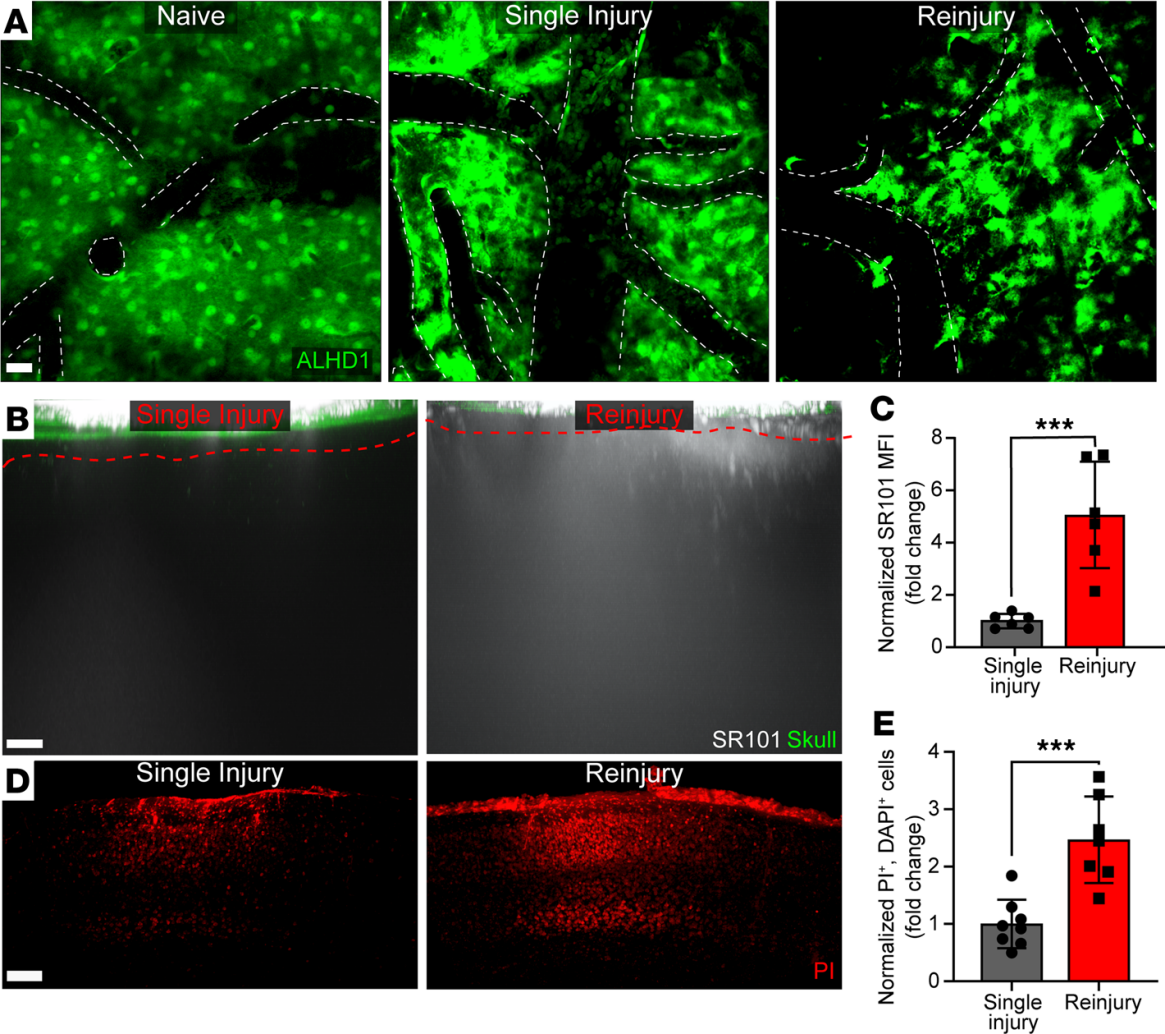
Reinjury promotes degradation of the glia limitans superficialis. (**A**) Representative *xy* maximally projected *Z* stacks (40 μm in depth) show ALDH1^+^ astrocytes (green) in *Aldh1*^CreER/+^ Stop^fl/fl^ TdTomato naive, single-injury, and reinjury mice. All injury mice were sacrificed at 30 hours after initial mTBI. White dotted lines denote the vasculature. Images are representative of 4 independent mice per group. Scale bar: 20 μm. (**B**) Representative *xz* maximally projected *Z* stacks (300 μm in depth) of SR101 leakage (white) applied transcranially through the skull (green). Glia limitans superficialis depicted as red-dashed line. Scale bar: 20 μm. (**C**) Quantification of SR101 leakage by mean fluorescent intensity (MFI). (**D**) Representative *xy* maximal projection *Z* stacks (100 μm in depth) shows propidium iodide–labeled (PI-labeled) dead cells (red). Scale bar: 100 μm. (**E**) Quantification of cell death by selecting only PI^+^DAPI^+^ cells. Each symbol in **C** and **E** depicts an individual mouse. Bar graphs represent 2 independent pooled experiments with 3–5 mice per group per experiment. Data were normalized to average single injury mouse per experimental day and displayed as the mean fold change ± SD with *P ≤ 0.05 and ***P ≤ 0.001 (2-tailed Student’s *t* test).

To understand breakdown and inflammation of the glia limitans superficialis after repetitive head injury, we developed a 24-hour reinjury model intended to reflect secondary injury to the region of the meninges and neocortex encountered 24 hours after primary injury. Mice receiving a mTBI as previously described (13, 14) at 0 hours and again at 24 hours were evaluated at 30 hours after the initial injury. Briefly, a meningeal compression injury was induced by thinning a small area of the skull bone (1 × 1 mm) to a thickness of 20–30 μm. The thinned skull was then depressed, resulting in an mTBI. Skull depression was repeated 24 hours later for the reinjury paradigm. These reinjured mice were compared at the same time point with mice that only received a single mTBI at 0 hours. We initially examined the structural integrity of the glia limitans superficialis in *Aldh1*^CreER/+^ Stop^fl/fl^ TdTomato mice fed tamoxifen chow for 3 weeks to induce expression of *Aldh1* — a promoter active in nearly all adult astrocytes (15). Two-photon imaging through the thinned skull window of uninjured reporter mice revealed a contiguous layer of surface-associated astrocytes comprising the glia limitans superficialis (Figure 1A). By contrast, a single mTBI examined at 30 hours after injury caused notable disruptions in the structural integrity of this barrier (Figure 1A). The breach in integrity was even more profound after a second mTBI at 24 hours (Figure 1A). Reinjury led to death of surface-associated astrocytes, evidenced by the absence of *Aldh1*^CreER/+^ Stop^fl/fl^ TdTomato signal in regions of the glia limitans superficialis.

To quantify the functional integrity of the glia limitans superficialis, we applied a low–molecular weight fluorescent dye (SR101) transcranially. When the glia limitans superficialis is intact, SR101 localizes primarily to the meninges (13). However, breach of this structure results in passage of SR101 into the neocortex, which can be quantified by 2-photon microscopy (13, 16). Using this assay, we determined that reinjury significantly increased glia limitans superficialis permeability relative to a single injury, allowing passage of more SR101 into the underlying brain parenchyma (Figure 1, B and C). This was associated with a marked increase in neocortical cell death, as revealed by quantification of transcranial propidium iodide staining (Figure 1, D and E). Further examination of the neocortical lesion after reinjury revealed a decrease in nucleated cells (Supplemental Figure 1, A and B; supplemental material available online with this article; https://doi.org/10.1172/jci.insight.149229DS1), NeuN^+^ neurons (Supplemental Figure 1, C and D), Iba1^+^ myeloid cells (Supplemental Figure 1, E and F), ALDH1^+^ astrocytes (Supplemental Figure 1, G and H), and APC^+^ oligodendrocytes (Supplemental Figure 1, I and J) — but not CD31^+^ endothelial cells (Supplemental Figure 1, K and L). These data demonstrate that the glia limitans superficialis is further breached following a second mTBI, which leads to enhanced permeability and indiscriminate parenchymal cell death.

### Microglia reinforce the glia limitans superficialis after primary but not secondary mTBI.

**Figure 2:**
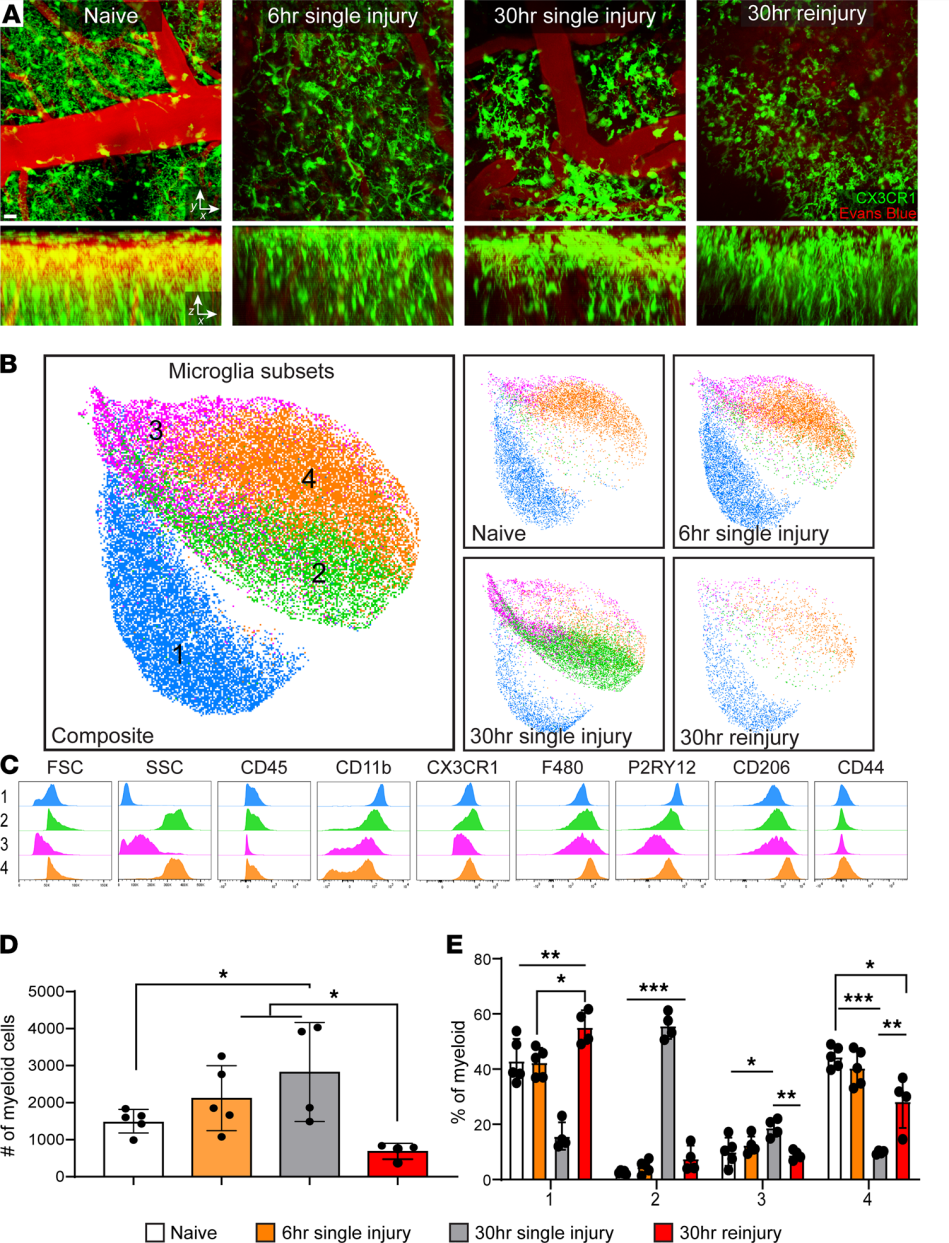
Reinjury promotes loss of microglia. (**A**) Representative *xy* (top) and *xz* (bottom) maximally projected *Z* stacks (180 μm in depth) show CX3CR1^gfp/+^ myeloid cells (green) and vasculature labeled with Evans blue (red) in *Cx3cr1*^gfp/+^ reporter mice without injury, with single injury, or with reinjury. See corresponding Supplemental Video 1. Images are representative of 4 independent mice per group. Scale bar: 20 μm (top, *xy*) and 20μm (bottom, *xz*). (**B**) Concatenated UMAPS generated from high-dimensional flow cytometric analysis (gating shown in Supplemental Figure 1A) of 2 mm cortical punch biopsies from the above groups show 4 different subsets of microglia identified as populations 1 (blue), 2 (green), 3 (pink), and 4 (orange). Dots depict cellularity. (**C**) Histograms show the relative expression of FSC-area (FSC-A), SSC-A, CD45, CD11b, CX3CR1, F480, P2RY12, CD206, and CD44 on composite, concatenated microglia populations 1 (blue), 2 (green), 3 (pink), and 4 (orange). (**D**) Quantification of microglia total per experimental group. (**E**) Quantification of the frequency of each microglia subtype per experimental group. Each symbol in **D** and **E** depicts an individual mouse. Bar graphs are representative of 2 independent experiments with 4–5 mice per experimental group. Data are displayed as mean ± SD with *P≤ 0.05, **P ≤ 0.01, and ***P ≤ 0.001 using 1-way ANOVA with Fisher’s LSD test (**D**) and multiple *t* tests with Holm-Sidak multiple comparisons test (**E**).

We demonstrated previously that microglia play an important role in fortifying the glia limitans superficialis after a single mTBI by projecting processes that seal gaps between surface-associated astrocytes (13). Inhibition of this neuroprotective microglia response resulted in enhanced leakage of the glia limitans superficialis. Given the importance of microglia in maintaining glia limitans integrity, we set out to compare microglia dynamics in *Cx3cr1*^gfp/+^ mice (17) after single and repeat mTBI using intravital 2-photon microscopy. Imaging of *Cx3cr1*^gfp/+^ mice revealed, as expected, that microglia project processes to the glia limitans superficialis 6 hours after a primary mTBI, and they were found to aggregate along this structure by 30 hours (Figure 2A and Supplemental Video 1). Conversely, reinjured mice at 30 hours after primary injury showed a reduced density of *Cx3cr1*^gfp/+^ cells along the glia limitans superficialis, suggestive of cell death (Figure 2A and Supplemental Video 1).

To evaluate the composition of the resident myeloid compartment in more detail, we performed high-parameter flow cytometric analyses on small punch biopsies of the damaged neocortex and glial limitans. Examination of the flow cytometric data using Uniform Manifold Approximation and Projections (UMAPs) revealed 4 different subsets of microglia that could be distinguished via forward scatter (FSC), side scatter (SSC), CD45, CD11b, CX3CR1, F4/80, P2RY12, and CD206 (Figure 2, B and C, and Supplemental Figure 2A). While microglial phenotypes were comparable between naive and mTBI mice at 6 hours after injury, a notable shift was observed at 30 hours in single-injury mice (Figure 2, B–E, and Supplemental Figure 2, A and B). A distinct subset of microglia (subpopulation 2) emerged at this time point that was larger (high FSC), more granular (high SSC), and more activated (high CX3CR1, F4/80, and low P2RY12 expression) than naive microglia (subpopulation 1) found predominantly in uninjured mice (Figure 2, B, C, and E; Supplemental Figure 2B; and Supplemental Figure 3). Importantly, this subpopulation of activated microglia was almost completely lost after reinjury (Figure 2, B–E; Supplemental Figure 2B; and Supplemental Figure 3). In fact, reinjured mice showed a numerical loss in all microglia subpopulations (Figure 2, B–E; Supplemental Figure 2B; and Supplemental Figure 3), consistent with the decreased cellularity observed in *Cx3cr1*^gfp/+^ mice by 2-photon microscopy (Figure 2A and Supplemental Video 1). These data suggest that neuroprotective microglia generated following a single mTBI are lost following reinjury. This conclusion is further supported by microglia depletion studies using the CSF-1R inhibitor PLX3397. Microglia depletion with PLX3397 increased permeability of the glia limitans superficialis after single mTBI (as expected) but had no impact on permeability in reinjury mice (Supplemental Figure 4, A–D).

### Reinjury generates a robust peripheral myelomonocytic cell response.

**Figure 3:**
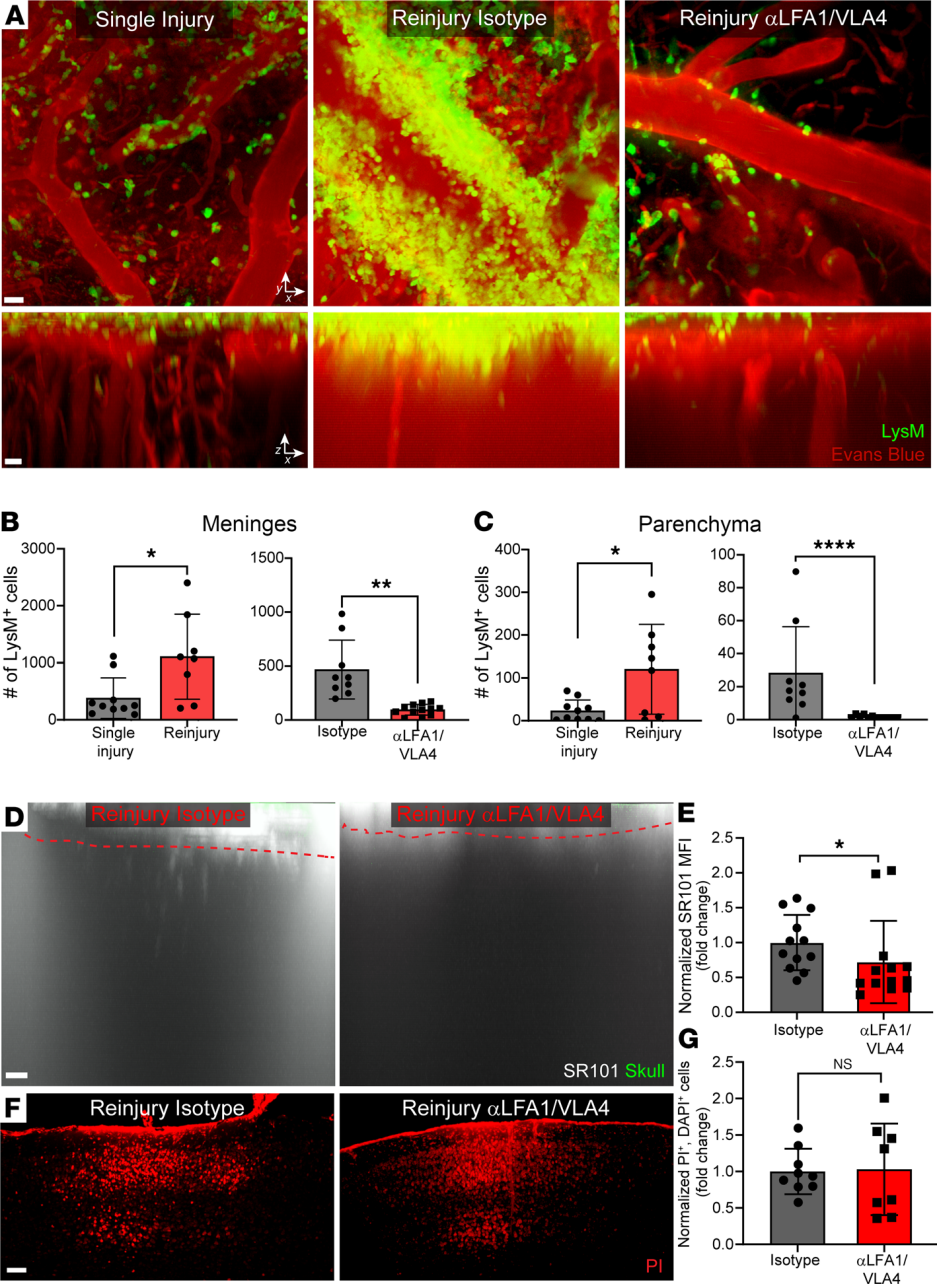
Reinjury promotes LFA-1/VLA-4–dependent recruitment of peripheral myelomonocytic cells. (**A**) Representative *xy* (top) and *xz* (bottom) maximally projected *Z* stacks (180 μm in depth) show LysM-GFP^+^ myelomonocytic cells (green) and vasculature labeled with Evans blue (red) in *LysM*^gfp/+^ reporter mice with a single injury or with reinjury and treated with isotype control or αLFA-1/VLA-4 antibodies. See corresponding Supplemental Video 2. Images are representative of 8 independent experiments with 2–4 mice. Scale bars: 40 μm (top, *xy*) and 20 μm (bottom, *xz*). (**B**) Quantification of total LysM-GFP^+^ myelomonocytic cells in the meninges. (**C**) Quantification of total myelomonocytic cells in the parenchyma. Bar graphs are representative of 8 independent experiments with 1–4 mice per group. (**D**) Representative *xz* maximally projected *Z* stacks (300 μm in depth) of SR101 leakage (white) applied transcranially through the skull (green) in reinjury mice treated either with isotype or αLFA-1/αVLA-4 antibodies. Glia limitans superficialis depicted as red dashed line. Scale bar: 40 μm. (**E**) Quantification of SR101 leakage by MFI. (**F**) Representative *xy* maximally projected *Z* stacks (100 μm in depth) show PI-labeled dead cells (red) in reinjury mice treated either with isotype or αLFA-1/αVLA-4 antibodies. Scale bar: 100 μm. (**G**) Quantification of cell death by selecting only PI^+^DAPI^+^ cells. (**B**, **C**, **E**, and **G**) Bar graphs represent 2–4 independent**,** pooled experiments with 2–5 mice per group per experiment. Each symbol depicts an individual mouse. (**B** and **C**) Data represent raw myelomonocytic cell counts. (**E** and **G**) Data were normalized to average reinjury isotype per experimental day. All data in **B**, **C**, **E**, and **G** are displayed as the mean fold change ± SD with *P ≤ 0.05, and **P ≤ 0.01 using 2-tailed Student’s *t* test.

In addition to microglia, peripheral myelomonocytic cells also respond to mTBI, typically within hours (13). We evaluated the recruitment of myelomonocytic cells in relation to primary and secondary injury of the glia limitans superficialis by imaging lysozyme GFP (*LysM*^gfp/+^) reporter mice (18). Two-photon imaging showed that a single mTBI promoted recruitment of LysM^gfp/+^ myelomonocytic cells to the meninges at 30 hours after injury, but few of these cells entered the brain parenchyma (Figure 3, A–C, and Supplemental Video 2). By contrast, reinjury markedly increased myelomonocytic cell recruitment to the meninges, as well as localization to the parenchyma (Figure 3, A–C, and Supplemental Video 2). To determine if enhanced recruitment of myelomonocytic cells contributed to breakdown of the glia limitans superficialis following reinjury, we developed an approach to block recruitment of these cells. This was accomplished by i.p. administering antibodies against the adhesion molecules LFA-1 and VLA-4 at 6 and 22 hours after single injury. LysM^gfp/+^ mice were then reinjured at 24 hours and imaged by 2-photon microscopy at 30 hours. Relative to isotype control antibody–treated mice, αLFA-1/VLA-4 significantly reduced recruitment of peripheral myelomonocytic cells to the meninges and brain parenchyma in reinjured mice (Figure 3, A–C, and Supplemental Video 2). This treatment also modestly improved integrity of the glia limitans superficialis, as measured by leakage of transcranial SR101 (Figure 3, D and E), but it did not reduce cell death in the brain parenchyma (Figure 3, F and G). Collectively, these data indicate that reinjury enhances LFA-1/VLA-4–dependent recruitment of peripheral myelomonocytic cells to the CNS that contribute partly to breakdown of the glia limitans superficialis.

### Reactive oxygen species generated after reinjury break down the glia limitans.

**Figure 4:**
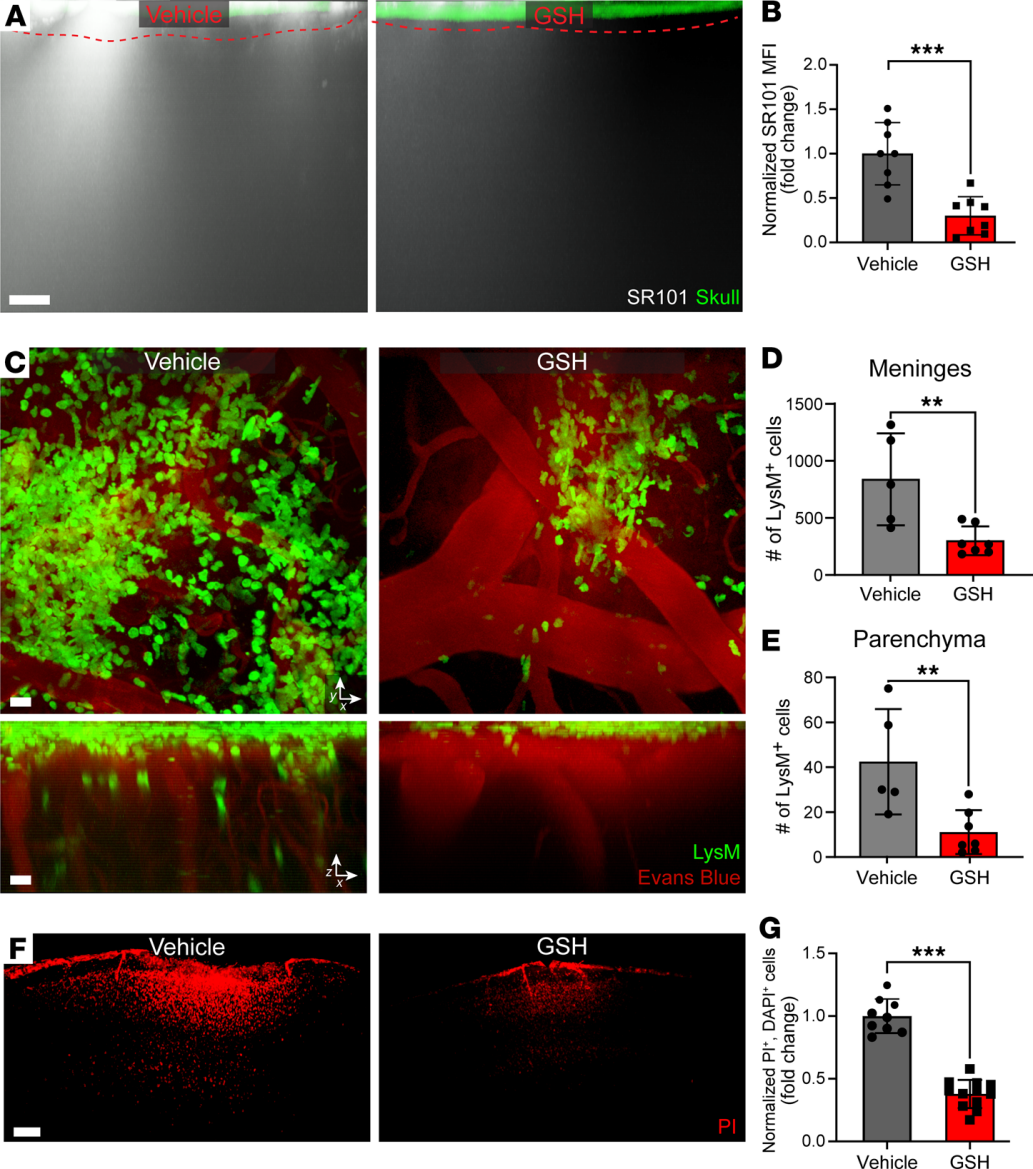
Free radicals contribute to glia limitans breakdown, inflammation, and cortical cell death following reinjury. (**A**) Representative *xz* maximally projected *Z* stacks (300 μm in depth) of SR101 leakage (white) applied transcranially through the skull (green) in vehicle reinjury and 10 mM GSH reinjury. Glia limitans depicted as red dashed line. Scale bar: 40 μm. (**B**) Quantification of SR101 leakage by MFI. (**C**) Representative *xy* (top) and *xz* (bottom) maximally projected *Z* stacks (180 μm in depth) show LysM-GFP^+^ myelomonocytic cells (green) and vasculature labeled with Evans blue (red) in reinjured *LysM*^gfp/+^ reporter mice treated with either vehicle (PBS) or glutathione (GSH). See corresponding Supplemental Video 3. Images are representative of 2 independent experiments with 1–2 mice per group. Scale bars: 40 μm (top, *xy*) and 40 μm (bottom, *xz*). (**D**) Quantification of total myelomonocytic cells in the meninges. (**E**) Quantification of total myelomonocytic cells in the parenchyma. (**F**) Representative *xy* maximally projected *Z* stacks (100 μm in depth) show PI-labeled dead cells (red) in vehicle- or GSH-treated reinjury mice. Scale bar: 200 μm. (**G**) Quantification of cell death by selecting only PI^+^DAPI^+^ cells. Bar graphs represent 2 (**B** and **G**) to 3 (**D** and **E**) independent, pooled experiments with 2–5 mice per group per experiment. Each symbol in **B**, **D**, **E**, and **G** depicts an individual mouse. (**B** and **G**) Data were normalized to average reinjury vehicle per experimental day. (**D** and **E**) Data represent raw myelomonocytic cell counts. All data in **B**, **D**, **E**, and **G** are displayed as the mean fold change ± SD with **P ≤ 0.01 and ***P ≤ 0.001 using 2-tailed Student’s *t* test.

To define the mediator of glia limitans breakdown after repeat injury, we turned our attention to what myelomonocytic cells and other distressed residents produce. Reactive oxygen species (ROS) are generated following brain injury and are produced by many different cell populations, including myelomonocytic cells (19). We observed diffuse ROS staining in the meninges both inside and outside of LysM-GFP^+^ myelomonocytic cells, supporting the notion that, after a second mTBI, multiple cell types produce free radicals (Supplemental Figure 5). We, therefore, evaluated the role of ROS in damaging the glia limitans superficialis by treating mice with glutathione (GSH) (a free radical scavenger) immediately following reinjury at 24 hours. Two-photon imaging of mice at 30 hours revealed that GSH significantly reduced breakdown of the glia limitans superficialis in reinjured mice; this was evidenced by decreased SR101 leakage (Figure 4, A and B) and preservation of surface associated astrocytes (Supplemental Figure 6), as well as SSC^hi^ microglia that were only observed in the single injury group (Supplemental Figure 7). Interestingly, GSH treatment also reduced recruitment of LysM^+^ myelomonocytic cells in response to reinjury (Figure 4, C–E, and Supplemental Video 3) and markedly decreased cell death in the brain parenchyma (Figure 4, F and G). These data demonstrate that free radicals are major contributors to destruction of the glia limitans superficialis and brain parenchyma after repeat mTBI.

## Discussion

Although decades of research have been dedicated to understanding blood brain barrier (BBB) destruction following TBI, little is known about mechanisms that inflame and break down the glia limitans superficialis, despite its crucial role in protecting the CNS parenchyma. Here, we highlight the glia limitans superficialis as an important barrier surface that fails following single and repeat mTBI, allowing fluids and mediators from the meninges to enter the brain parenchyma. Notably, we demonstrate that a second brain injury promotes further deterioration of the glia limitans superficialis by harming surface-associated astrocytes, which decreases barrier integrity and enhances cortical cell death relative to a single injury. Moreover, we observed that microglia, which normally help stabilize the glia limitans superficialis after a single mTBI (13), no longer retain their barrier-promoting properties and die after repeat injury. This is associated with a massive influx of myelomonocytic cells that invade the reinjured meninges and parenchyma in an LFA-1/VLA-4–dependent manner. Inhibition of this recruitment modestly preserved glia limitans integrity but failed to reduce cortical cell death. These data, coupled with diffuse ROS staining in the lesion, suggests that cells, in addition to invading myelomonocytic cells, are responsible for death of the glia limitans and underlying parenchyma. Importantly, therapeutic scavenging of free radicals with GSH dramatically preserved glia limitans integrity and barrier function after reinjury. GSH also markedly reduced cortical cell death and myelomonocytic cell invasion. Collectively, these data demonstrate that the glia limitans superficialis is an integral, protective barrier surface that undergoes ROS-mediated breakdown following repeat mTBI.

The glia limitans perivascularis, which surrounds perivascular spaces in the CNS parenchyma, has long been associated with BBB breakdown during neurodegenerative, autoimmune, and infectious diseases (12, 20–22). However, few studies have focused on the glia limitans superficialis that covers the entire brain and spinal cord parenchyma. Like the traditional BBB, we observed that the glia limitans superficialis is susceptible to deterioration and leakage after single and repeat mTBI. The extent of injury is more profound after reinjury and is associated with ROS-dependent destruction of surface-associated astrocytes and cortical cells. This response is distinct from a single focal mTBI, during which a modest breach of the glia limitans superficialis is resolved by underlying microglia that project processes in a purinergic receptor–dependent manner and seal gaps between individual surface-associated astrocytes (13, 23). Microglia processes can similarly stabilize the BBB and limit the extent of leakage following cerebrovascular injury (24, 25). These reactions transpire within minutes of injury and depend on the well-described ability of microglia to detect extracellular ATP released from astrocytes via purinergic receptors like P2RY12 and P2RX4 (26, 27). Consistent with this barrier-preserving role, we demonstrated that depletion of microglia with a CSF-1R inhibitor increased leakage of the glia limitans superficialis following a single injury. By contrast, microglia depletion had no impact on barrier leakage following reinjury. At the time of reinjury, microglia had converted from their homeostatic state into amoeboid phagocytes and were likely unable to dedifferentiate and respond to the secondary insult by projecting barrier-sealing processes. In addition, many microglia as well as astrocytes died following reinjury, making the glia limitans superficialis even more prone to leakage, but both cell populations were preserved by GSH treatment. It will be important in future studies to determine when or if microglia return to a naive state along the glia limitans superficialis after brain injury, poised once more to respond to mechanical injury. It is conceivable that these cells become imprinted with new properties that make them less capable of responding to future insults (28, 29). Given the well-described dialogue between microglia and astrocytes (30), it will also be important to determine how surface-associated astrocytes remodel along the glia limitans superficialis after primary and secondary brain injuries.

Single-cell sequencing has identified many subtypes of microglia, including injury-responsive microglia, that develop in response to toxin-induced lesions and TBI (31, 32). By performing high-parameter flow cytometry on cortical biopsies from mTBI mice, we identified 4 subpopulations of microglia, including one (subpopulation 2) that appeared predominantly at 30 hours following single injury. These microglia downregulated P2RY12 — a sign of activation (33, 34) — and upregulated CX3CR1 and F4/80. They were also large (high FSC) and granular (high SSC), suggesting conversion into a phagocyte. This conclusion was supported by the amoeboid cells observed beneath the injured glia limitans superficialis in Cx3CR1^gfp/+^ mice at 30 hours by 2-photon microscopy. Importantly, reinjury resulted in the near complete loss of this microglia subtype, signifying the end of their contribution to the injury response. We postulate that the differentiation state and susceptibility of microglia to death after a single brain injury makes the glia limitans superficialis especially vulnerable to collapse upon reinjury.

In moderate to severe TBI models, infiltration of inflammatory myelomonocytic cells has been associated with worse outcomes (35). However, myelomonocytic cells paradoxically also play an important role in repair after CNS injuries, including TBI (10, 36). One possible explanation for this discrepancy is that myelomonocytic cells can contribute to CNS damage and repair, depending on the time point after injury. For example, it was revealed in a model of cerebrovascular injury that robust myelomonocytic cell extravasation early after injury caused vascular breakdown and edema, but these same cells were required at later time points after injury to promote vascular repair (25). A similar scenario may unfold following mTBI. Myelomonocytic cells are required for meningeal repair after a single mTBI (10); however, we observed that reinjury massively increases LFA-1/VLA-4–dependent recruitment of myelomonocytic cells that contributes to increased glia limitans superficialis leakage. This leakage could result from production of free radicals by myelomonocytic cells and/or their ability to breach the integrity of blood vessels as they extravasate (12, 37). Recruited peripheral immune cells (e.g., myelomonocytic cells) and stressed resident cells (e.g., microglia) are known producers of free radicals, which have long been implicated in TBI pathophysiology (19, 38, 39). In fact, studies in rodents and humans have shown that systemic ROS reduction either by scavenging or restricting production is neuroprotective and improves neurologic function following brain injury (40–43). Importantly, our data demonstrate that administration of the ROS scavenger GSH decreases cortical cell death and is neuroprotective even after a second head injury. Therapeutic administration of GSH also showed a remarkable ability to preserve the glia limitans superficialis and reduce myelomonocytic cell recruitment to both the meninges and parenchyma. These data indicate that free radicals, likely produced by several different cell types after mTBI, play a major role in harming the glia limitans superficialis and underlying brain parenchyma. Antioxidants like GSH should, thus, be considered as an acute therapeutic intervention for both single and repeat mTBI.

In conclusion, our study provides insights into the breakdown of the glia limitans superficialis after brain injury, and it addresses how resident and peripheral myeloid cells respond to multiple injuries encountered along this structure. In humans, the glia limitans superficialis is especially susceptible to repetitive injuries (6), and it is therefore important to uncover more insights into how this barrier is maintained and repaired after brain injury. While innate myeloid cells orchestrate different aspects of the response to glia limitans injury, the major role of ROS in the pathophysiology single and repeat mTBI should not be ignored. Free radicals contribute to destruction of the glia limitans superficialis and cortex after brain injury, and GSH is a remarkably effective therapeutic that can be used to mitigate damage and inflammation. Future studies should focus on identifying other factors that help preserve and promote restoration of the glia limitans superficialis after brain injuries, especially those encountered repetitively.

## Methods

### Mice

C57BL/6J (B6), B6N.FVB-Tg(Aldh1l1-cre/ERT2)1Khakh/J (*Aldh1*^CreER/CreER^) (15), B6.129P-Cx3cr1^tm1Litt^/J (*Cx3cr1*^gfp/gfp^) (17), and B6.Cg-Gt(ROSA)26Sortm14(CAG-tdTomato)Hze/J (Stop^fl/fl^ TdTomato) (44) mice were obtained from the Jackson Laboratory. B6.LysM^gfp/+^ (*LysM*^gfp/+^) mice were provided by T. Graf (Albert Einstein College of Medicine, Bronx, New York, USA) (18). Strains *Cx3cr1*^gfp/gfp^ and *LysM*^gfp/gfp^ mice were crossed with B6 mice to generate heterozygous reporter mice used for experiments. *A1ldh1*^CreER/CreER^ mice were crossed with Stop^fl/fl^ TdTomato mice. The resultant F1 mice were fed tamoxifen chow (Envigo) for 2–3 weeks to induce expression of TdTomato in astrocytes. Mice were studied at ages ranging from 6 to 12 weeks.

### Skull thinning and mTBI

All brain injuries were performed as described previously (14). Mice were anesthetized by i.p. injection of ketamine (85 mg/kg; Ketaset), xylazine (13 mg/kg; AnaSed), and acepromazine (2 mg/kg; VET one) in PBS, and body temperatures were held at 37°C. An incision was made to expose the skull bone, and a metal bracket was secured on the skull bone over the barrel cortex (2.5 mm from bregma × 2.5 mm from sagittal suture). A 1 × 1 mm cranial window was quickly thinned over 1–2 minutes using a dental drill with a 0.5 mm burr to a thickness of ~20–30 μm. Once thinned, a microsurgical blade was used to lightly compress the skull bone. For survival surgeries, the incision was closed with sutures, and mice were injected i.p. with Buprenex (0.1 mg/kg; Buprenex) for pain management, as well as Antisedan (1 mg/kg; Revertidine) for anesthetic reversal. For reinjury studies, mice were anesthetized and prepped as described above, followed by additional compressions with the microblade at 24 hours after primary injury.

### Blocking and antagonism studies

#### LFA-1/VLA-4 blockade.

To block cell adhesion to the endothelium of peripheral immune cells, we used 500 μg anti–LFA-1 (clone M17/4, BE0006) and 500 μg anti–VLA-4 (clone PS/2, BE0071) purchased from BioXcell. Rat IgG from BioXcell was used as an isotype control (catalog BE0090). LFA-1/VLA-4 blockade or isotype control was given i.p. at 6 hours and 22 hours after single injury.

#### ROS antagonism.

ROS were scavenged by i.p. injecting GSH (3.07 mg/mL, G4251-5G, MilliporeSigma) dissolved in 200 μL PBS and pH corrected to ~6.5 immediately following secondary injury. Vehicle was injected into the control mice.

#### Microglia depletion.

Microglia were depleted using the CSF-1R inhibitor, PLX3397 (A15520, Adooq Bioscience). Mice were fed PLX3397 formulated as a chow (300 mg/kg, Envigo) for a minimum of 14 days. Depletion efficiency was ~50% in the neocortex, which was determined by counting Iba1^+^ spots on immunohistochemically stained brain sections from untreated versus PLX3397-treated mice using Imaris version 9.6 image analysis software (Bitplane).

### Intravital 2-photon microscopy

mTBI and control mice were imaged using a Leica SP8 2-photon microscope equipped with an 8000-Hz resonant scanner, a 25× collar-corrected water-dipping objective (1.0 NA), a quad HyD external detector array, and a Mai Tai HP DeepSee Laser (Spectra-Physics) tuned to 905 nm. Three-dimensional time-lapse movies were captured as *Z* stacks. For blood vessel visualization, 100 μL of 1 mg/mL Evans blue (MilliporeSigma) dissolved in PBS was injected i.v. immediately following surgical preparation. For all imaging, artificial cerebral spinal fluid (aCSF) (Harvard Apparatus, 597316) was used to submerge the lens above the thinned skull.

#### ALDH1^CreER/+^x Stop^fl/fl^ TdTomato mice.

Surface associated astrocytes comprising the glia limitans superficialis were visualized using *ALDH1*^CreER/+^*x* Stop^fl/fl^ TdTomato mice. A 3-dimensional 40 μm *Z* stack (1 μm step size) was then captured through a thinned skull window of naive mice, as well as at 24 hours and 30 hours after initial injury in single and repeat mTBI mice. For GSH-treated mice, resultant image files were then analyzed using Imaris version 9.6 image analysis software. An ALDH1^+^ “surface” was created for each image and recorded.

#### Glia limitans leakage assay.

To assess the integrity of the glia limitans, 200 μL sulforhodamine 101 (SR101) (1 mM, S7635, Sigma-Aldrich) dissolved in PBS was applied to the skull for 12 minutes. SR101 was then washed several times with aCSF over a 5-minute period. A 3-dimensional 300 μm *Z* stack (1 μm step size) was then captured using the 2-photon microscope. *Z* stacks were started just above the skull bone. For quantification, *Z* stacks were cropped to 250 μm to remove the skull bone. The mean fluorescence intensity (MFI) of the channel corresponding to SR101 signal (562–650 nm) was then calculated using Imaris version 9.6 image analysis software. MFI data were normalized for each experiment by averaging of the single injury or nontreated group and calculating the fold increase for each individual sample relative to the average. Data were then expressed as a fold increase.

#### CX3CR1^gfp/+^ mice.

Myeloid cells were visualized using *CX3CR1*^gfp/+^ mice. Thirty-minute 3-dimensional 180 μm *Z* stacks (3 μm step size) were captured through a thinned-skull window in naive mice and following single and repeat mTBI using the 2-photon microscope.

#### LysM^gfp/+^ mice.

Following described single and repeat injury, *LysM*^gfp/+^ mice were imaged to visualize myelomonocytic cells. Three-dimensional 210 μm Z stacks (3 μm step size) were captured by 2-photon microscopy for 30 minutes at 30 hours after initial injury. Using Imaris version 9.6 image analysis software, the meninges and parenchyma were separately identified and analyzed. Based on dural and pial vasculature, the top 38–45 μm of each 210 μm *Z* stack was identified as the meningeal space. Underlying tissue was identified as the parenchyma and adjusted to a uniform 150 μm for quantification. For both the meninges and parenchyma, a 3-dimensional “surface” corresponding to LysM-GFP fluorescence was created and divided by the average size of a myeloid cell (assumed as 1400 m^3^) to calculate the total number of myelomonocytic cells in each compartment.

#### Amplex Red ROS detection assay.

To visualize ROS after mTBI, 200μL of Amplex Red Reagent (500 μM, A12222, Thermo Fisher Scientific) dissolved in aCSF was applied to the skull bone of LysM^gfp/+^ mice for 10 minutes. Amplex Red Reagent was then washed once with aCSF and then imaged immediately by 2-photon microscopy. A 3-dimensional 50 μm *Z* stack (1 m step size) was captured beginning a the thinned skull bone.

### IHC

#### Tissue preparation.

In total, 200 μL propidium iodide, a cell-membrane impermeable dye (1 mg/mL, P1304MP, Thermo Fisher Scientific), was applied to the thinned skull bone for 1 hour at 29 hours after initial injury. Mice then received an intracardiac perfusion with 4% paraformaldehyde (PFA). Heads were removed and incubated overnight at room temperature in 4% PFA. Brains were sectioned at thickness of 100 μm through the entire lesion using a Compresstome (Precisionary).

#### Dead cell detection and quantification.

Brain sections were well stained in 500 μL of PBS with 1:500 DAPI (1 mg/mL, D9542, Sigma-Aldrich) for 10 minutes before washing and mounting on charged glass slides. A single section with the highest density of propidium iodide staining was then imaged through entire *Z* plane using an Olympus FV1200 laser-scanning confocal microscope fitted with a 10× objective. Resultant image files were quantified using Imaris version 9.6 image analysis software (Bitplane). DAPI^+^ “surfaces” were created, and dead cells were enumerated as DAPI^+^ cells that colocalized with propidium iodide. Dead cells were normalized for each experiment by averaging the single injury or nontreated group and calculating the fold change for each individual sample relative to the average. Data were then expressed as a fold change.

#### Identification of dead cells.

Prior to IHC staining, sections were incubated in 500 μL PBS containing 0.1% Triton X-100 (AC215682, Thermo Fisher Scientific) and 3 drops of Background Buster (NB306, Innovex Biosciences) for 30 minutes. To visualize neurons (NeuN^+^) and microglia (Iba1^+^), brain sections were incubated with guinea pig anti-NeuN (1:500, ABN91, MilliporeSigma) and rabbit anti-Iba1 (1:500, 019-19741, Wako Chemicals) together. To label endothelial cells (CD31^+^) and oligodendrocytes (APC^+^), mice were first anesthetized and retro-orbitally injected with 50 μL CD31 Alexa Fluor 647 in PBS (1:500, 102516, BioLegend). Brain tissue was then prepared and sliced as described above. Afterward, the tissue was stained with a mouse anti–adenomatous polyposis coli (anti-APC) antibody (1:500, OP80, Sigma-Aldrich). ALDH1^+^ astrocytes were labeled endogenously in tamoxifen-treated *ALDH1^CreER/+^x Stop^fl/fl^* TdTomato mice. Brain sections from these mice were stained with anti-Iba1 antibody. All primary antibodies solutions were incubated overnight at 4°C on a shaker. The following morning sections were washed 3 times with PBS before incubating in 500 μL PBS containing 0.1% Triton X-100 for 2 hours with one of the following secondaries: goat anti–guinea pig Alexa Fluor 488 (1:500, A-11073, Thermo Fisher Scientific), donkey anti–rabbit Alexa Fluor 647 (1:500, A-31573, Thermo Fisher Scientific), or goat anti–mouse Alexa Fluor 488 (1:500, A-21131, Thermo Fisher Scientific). Finally, DAPI was added for 10 minutes as described previously, and sections were washed and mounted on charged glass slides. For NeuN-, Iba1-, intravascular CD31– (ivCD31-), and APC-stained tissue, a single brain section with the highest density of propidium iodide signal was imaged using an Olympus FV1200 laser-scanning confocal microscope fitted with a 20× objective (20 μm *Z* stack). As a control, the corresponding uninjured contralateral hemispheres were imaged, as well, using the same parameters. Resultant confocal images were then quantified using Imaris version 9.6 image analysis software. DAPI^+^ surfaces were created, and dead cells were defined as DAPI^+^ cells that colocalized with propidium iodide. To quantify individual cell types of interest (NeuN^+^, Iba1^+^, APC^+^, ALDH1^+^), a 3-dimensional “spot” corresponding to each respective cell type was created in Imaris. CD31^+^ cells were quantified by creating a 3-dimensional “surface” of the entire CD31^+^ signal. The density of each cell type was normalized per experiment by averaging of the cell count in the uninjured contralateral hemisphere and calculating the fold difference for each individual sample relative to the average. Data were then expressed as a fold change.

### Flow cytometry

#### Leukocyte isolation.

Naive mice, 6 hours post–single injury mice, and 30 hours post–single or reinjury mice were anesthetized and decapitated, and their brains were explanted. A 2 mm punch biopsy (MTP-33-34, Brain Tree Scientific) corresponding to the mTBI lesion (2.5 mm from bregma × 2.5 mm from sagittal suture) was then taken. Leukocytes were isolated by mashing the 2 mm brain biopsy through a 40 µm cell strainer (TS40, Alkali Scientific) and washed with PBS containing 2% FBS (FACS buffer). Samples were centrifuged at 500*g* for 5 minutes at 4°C in 50 mL conical tubes, and pellets were isolated. After resuspending cells in 1 mL FACS buffer, cells were passed through a 35 μm cell strainer (352235, Corning). An additional 1 mL of FACS buffer and 860 μL 90% Percoll (GE17-5445-01, Sigma-Aldrich) were passed through the cell strainer, resulting in a 30% Percoll solution. Samples were centrifuged for 15 minutes at 500*g* at 4°C. Myelin and other debris were removed by aspirating to final ~250 μL cellular pellet. Cells were then stained.

#### FACS staining.

Cell suspensions were washed and resuspended in 50 μL master staining mix containing FACS buffer, anti–mouse CD16/32 (1:200, 553141, BD Biosciences [BD]), and the following antibodies obtained from BioLegend (BL) or BD: CD11c AF488 (1:200, 117311, BL), Ly6C BB796 (1:200, custom-made, BD), I-A/I-E BV480 (1:450, 566088, BD), CD11b BV570 (1:100, 101233, BL), TCR β BV605 (1:100, 109241, BL), CD24 BV650 (1:300, 563545, BD), CX3CR1 BV711 (1:100, 149031, BL), P2RY12 PE (1:200, 848004, BL), Ter-119 AF647 (1:1000, 116218, BL), CD206 PE-Cy7 (1:400, 141720, BL), CD45 BUV395 (1:200, 564279, BD), Ly6G BUV650 (1:150, 740554, BD), CD19 BUV680 (1:100, custom-made, BD), CD44 BUV737 (1:1100, 612799, BD), CD115 APC (1:200, 135510, BL), and F/480 APC-R700 (1:500, 565787, BD). Cells were protected from light and incubated on ice for 15 minutes and at room temperature for 15 minutes, followed by washing. Dead cells were excluded by incubating cells with 50 μL LiveDead fixable Blue Cell Staining kit (1:500 diluted, L34962, Thermo Fisher Scientific) on ice for 10 minutes. Cells were then fixed in 10% formalin for 10 minutes, washed, and placed at 4°C overnight. Samples were acquired using a BD FACSymphony A-5 digital cytometer, and data were analyzed using FlowJo software version 10.

#### UMAP analysis.

Acquired samples were loaded into FlowJo software version 10 and cleaned using the FlowAI algorithm. Samples were compensated and gated as shown in Supplemental Figure 1A to identify resident myeloid cells. The phenograph and UMAP plug-ins in FlowJo were used to identify the different microglia subpopulations.

### Statistics

Analyses involving 2 or more groups were performed 2-tailed Student’s *t* test or 1-way ANOVA, respectively. For comparisons involving groups within groups, multiple *t* tests with Holm-Sidak multiple comparisons test were utilized. Data were normalized to the average of each experiment’s control group and displayed as fold change. Statistical significance was determined as *P* ≤ 0.05. All data are shown as the mean ± SD.

### Study approval

All mice were maintained in a closed breeding facility and were housed and treated in accordance with the IACUC at the NIH. 

## Author contributions

DM and HM designed the research. HM, AJ, NM, and PM performed experiments. HM analyzed the data. HM and DM wrote and edited the manuscript. DM supervised the project and contributed to data acquisition and analysis.

## Supplementary Material

Supplemental data

Supplemental video 1

Supplemental video 2

Supplemental video 3

## Version 1 08/24/2021

In-Press Preview

## Version 2 10/08/2021

Electronic publication
