# Supplementary material for: Glia limitans superficialis oxidation and breakdown promote cortical cell death after repetitive head injury
Source: JCI Insight. 2021 Oct 8;6(19):e149229. doi: 10.1172/jci.insight.149229 (PMC8525634; doi:10.1172/jci.insight.149229)
Supplement: Supplemental data [file jciinsight-6-149229-s185.pdf]

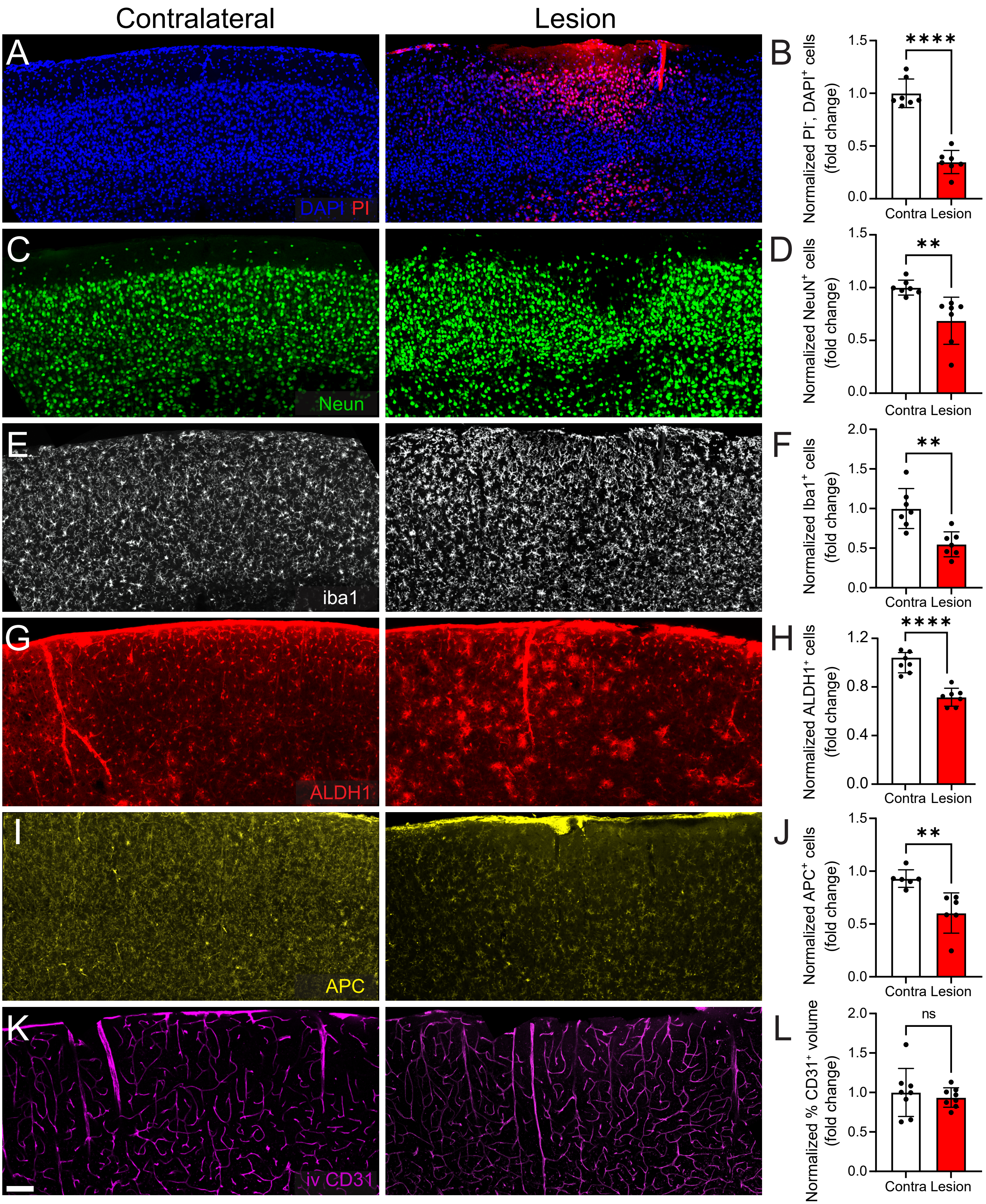

Contralateral
Lesion
A
DAPI PI
B
Normalized PI-, DAPI+ cells (fold change)
****
Contra Lesion
C
Neun
D
Normalized NeuN+ cells (fold change)
**
Contra Lesion
E
iba1
F
Normalized Iba1+ cells (fold change)
**
Contra Lesion
G
ALDH1
H
Normalized ALDH1+ cells (fold change)
****
Contra Lesion
I
APC
J
Normalized APC+ cells (fold change)
**
Contra Lesion
K
iv CD31
L
Normalized % CD31+ volume (fold change)
ns
Contra Lesion

Figure S1

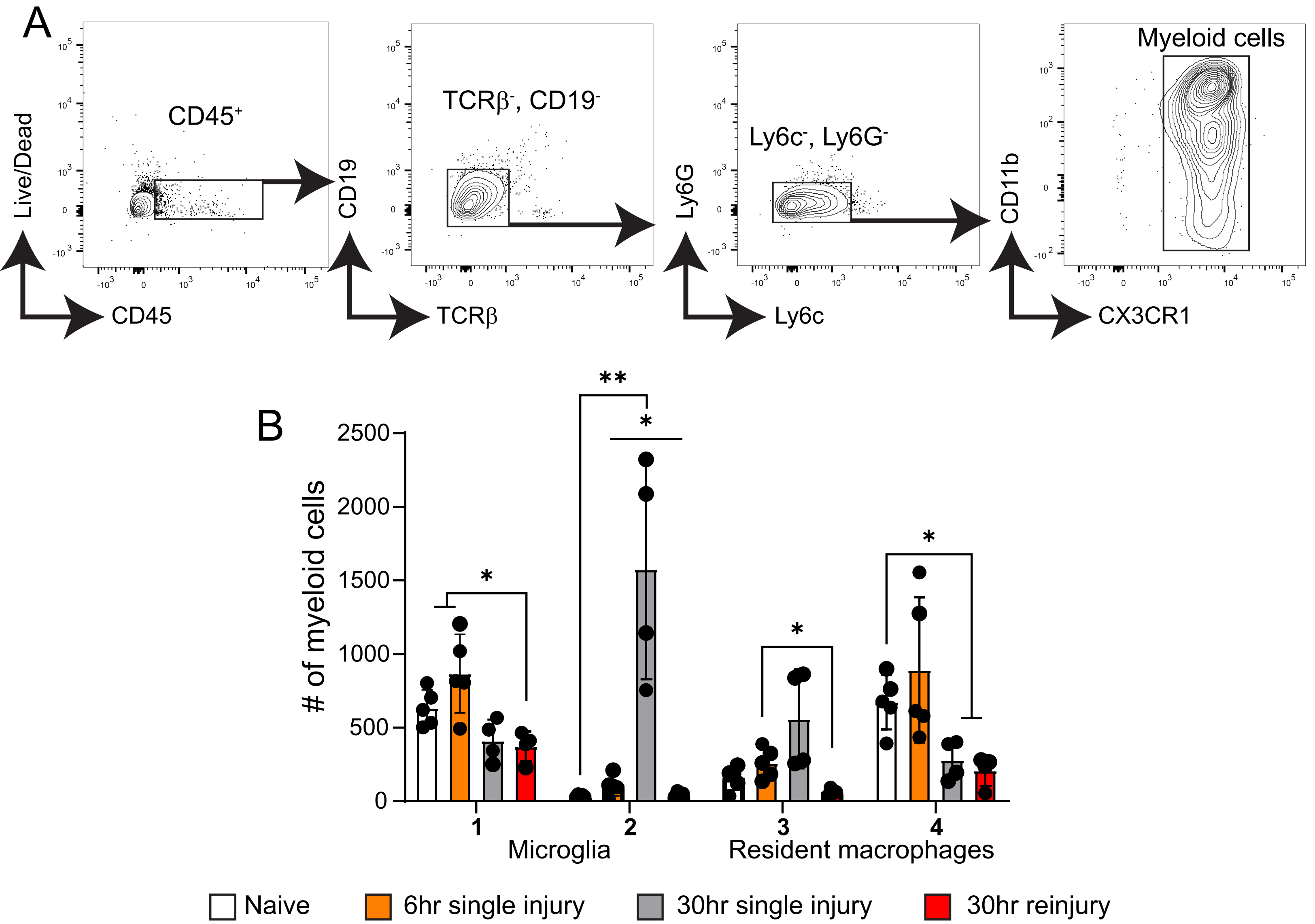
A
CD45+
Live/Dead
CD45
TCRβ-, CD19-
CD19
TCRβ
Ly6c-, Ly6G-
Ly6G
Ly6c
Myeloid cells
CD11b
CX3CR1
B
# of myeloid cells
2500
2000
1500
1000
500
0
**
*
*
*
*
1
2
3
4
Microglia
Resident macrophages
Naive
6hr single injury
30hr single injury
30hr reinjury

Figure S2

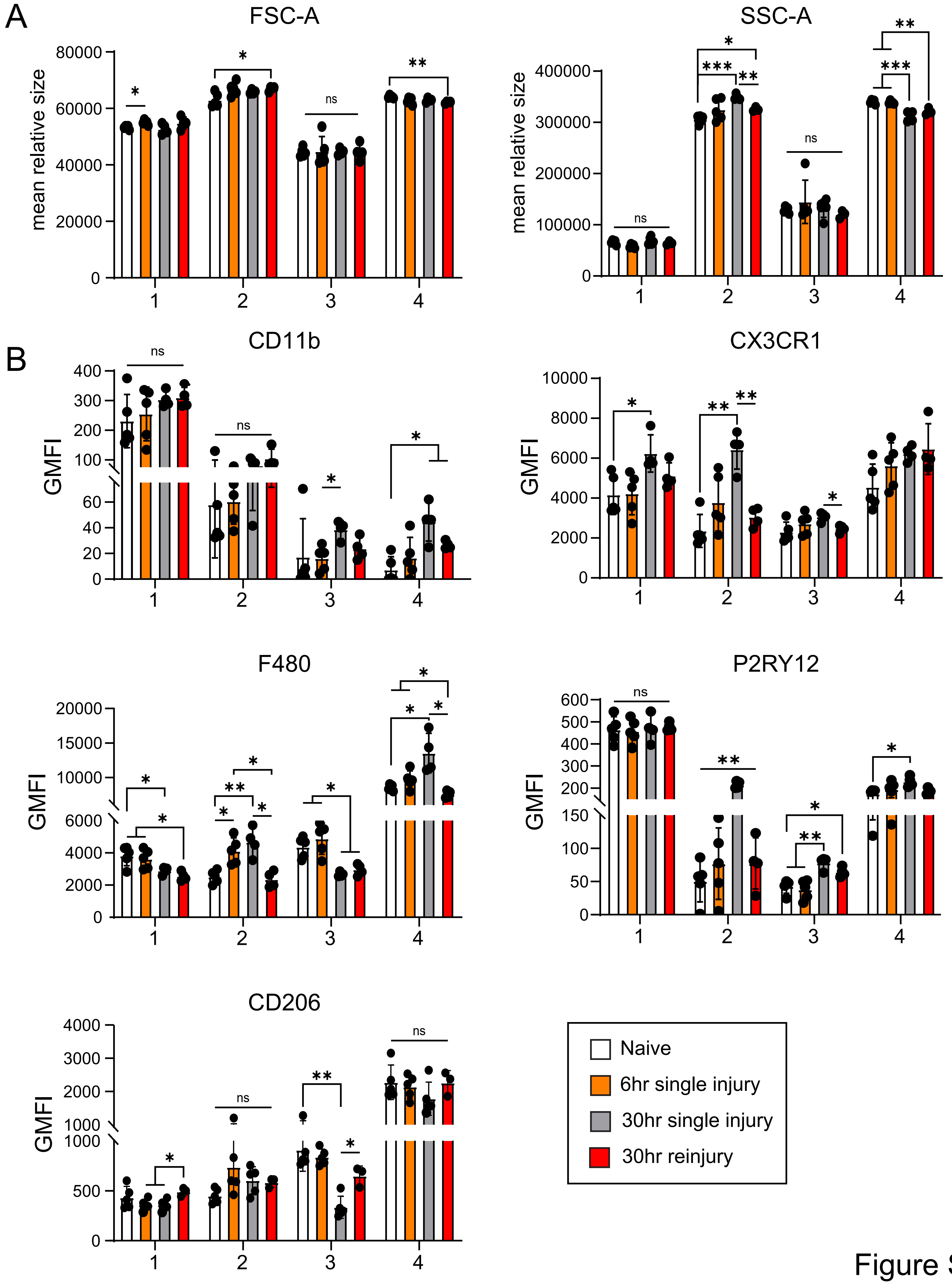
A
FSC-A
mean relative size
SSC-A
B
CD11b
GMFI
CX3CR1
F480
P2RY12
CD206
Naive
6hr single injury
30hr single injury
30hr reinjury

Figure S3

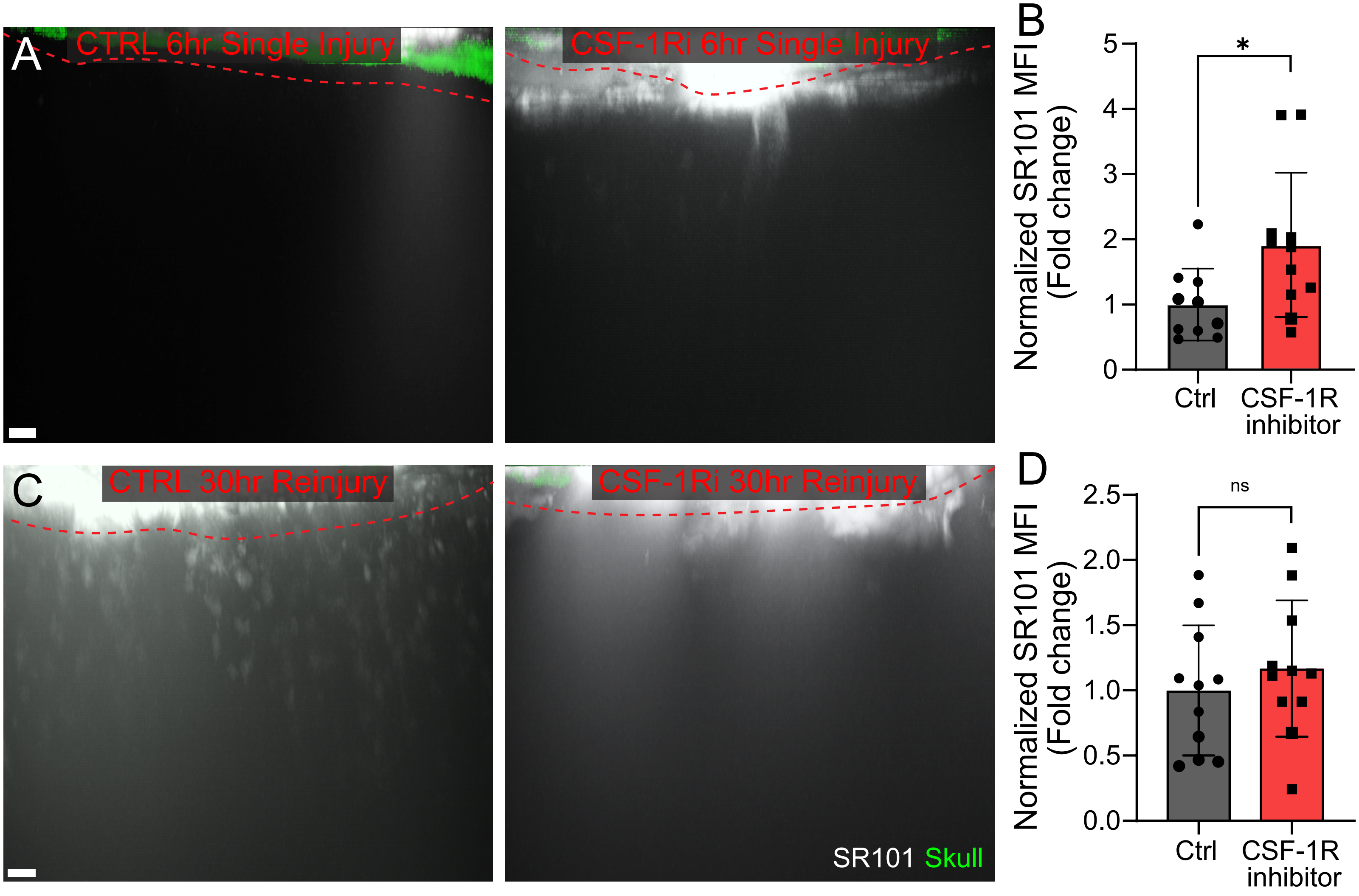
A
CTRL 6hr Single Injury
CSF-1Ri 6hr Single Injury
B
Normalized SR101 MFI (Fold change)
0
1
2
3
4
5
*
Ctrl
CSF-1R inhibitor
C
CTRL 30hr Reinjury
CSF-1Ri 30hr Reinjury
SR101 Skull
D
Normalized SR101 MFI (Fold change)
0.0
0.5
1.0
1.5
2.0
2.5
ns
Ctrl
CSF-1R inhibitor

Figure S4

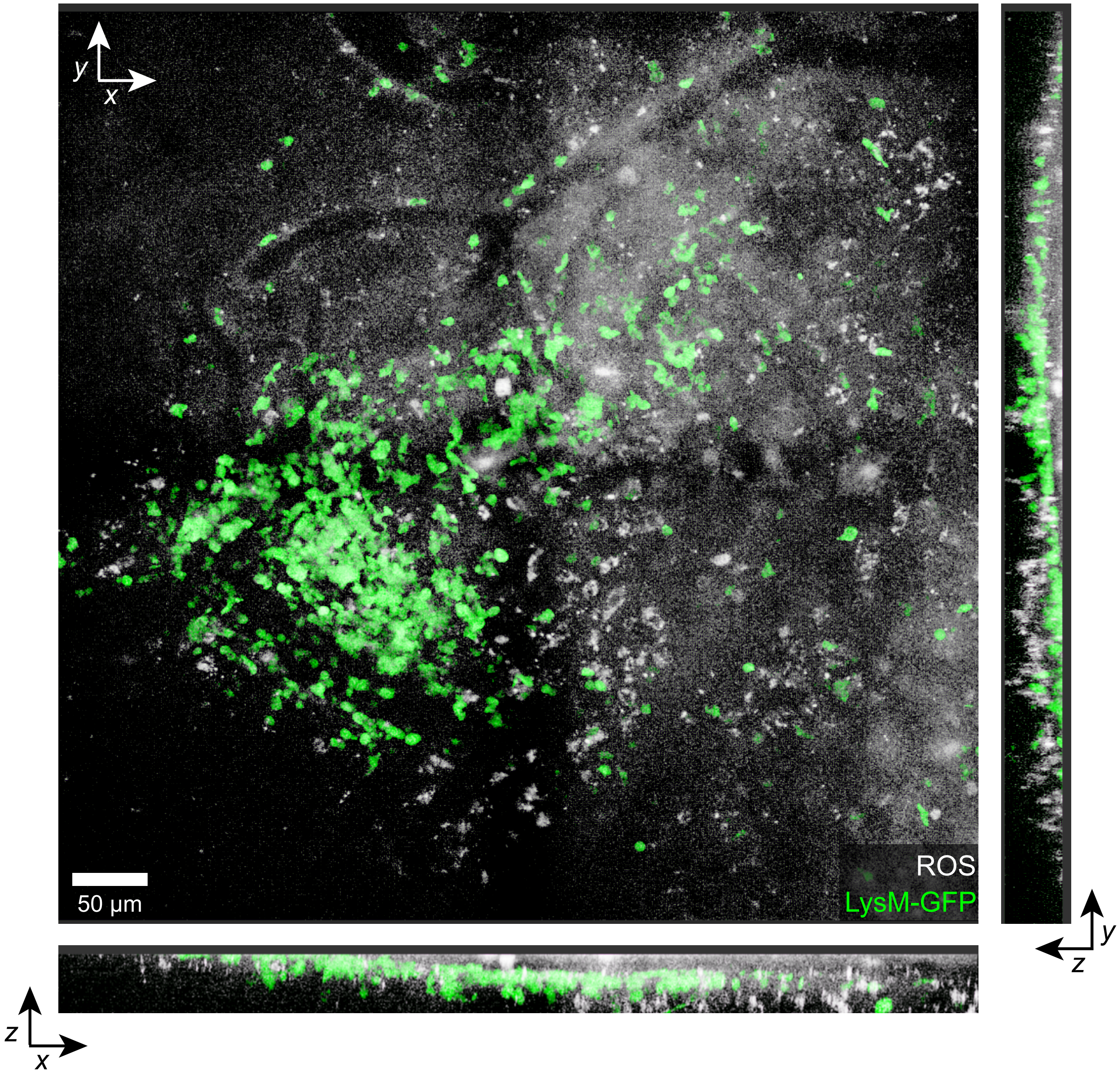

Figure S5

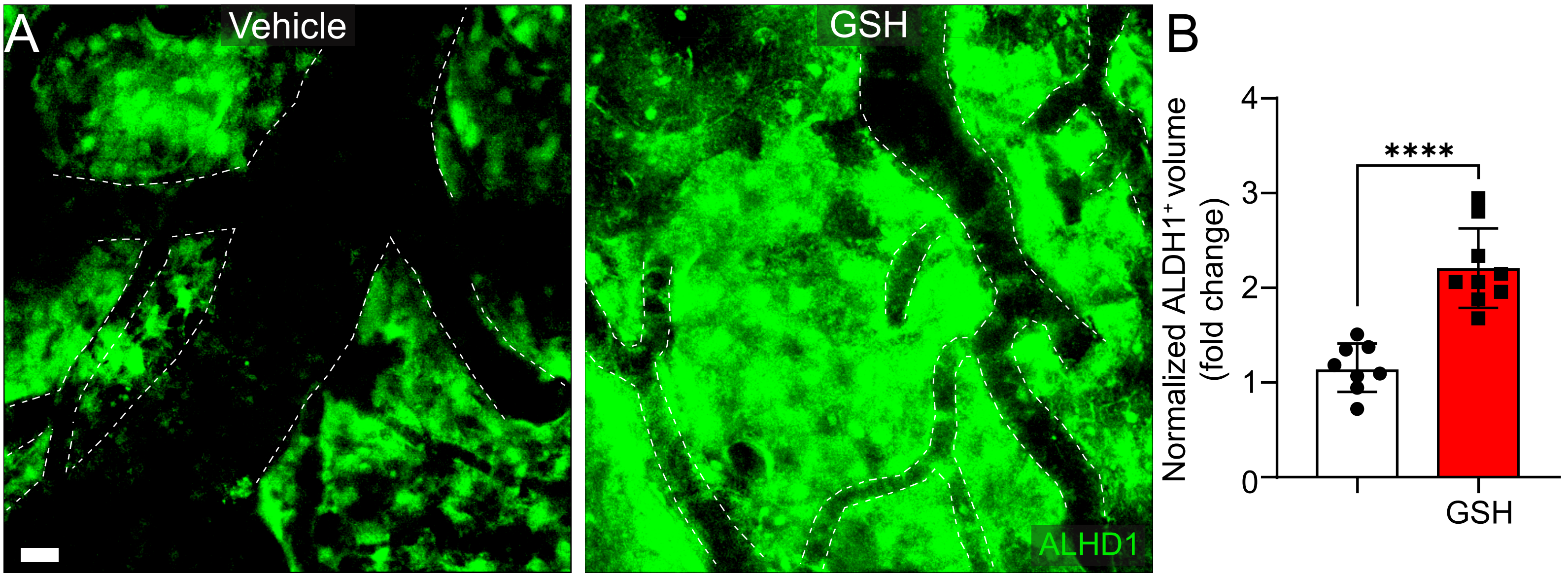
A
Vehicle
GSH
ALHD1
B
Normalized ALDH1$^+$ volume (fold change)
4
3
2
1
0
****
GSH

Figure S6

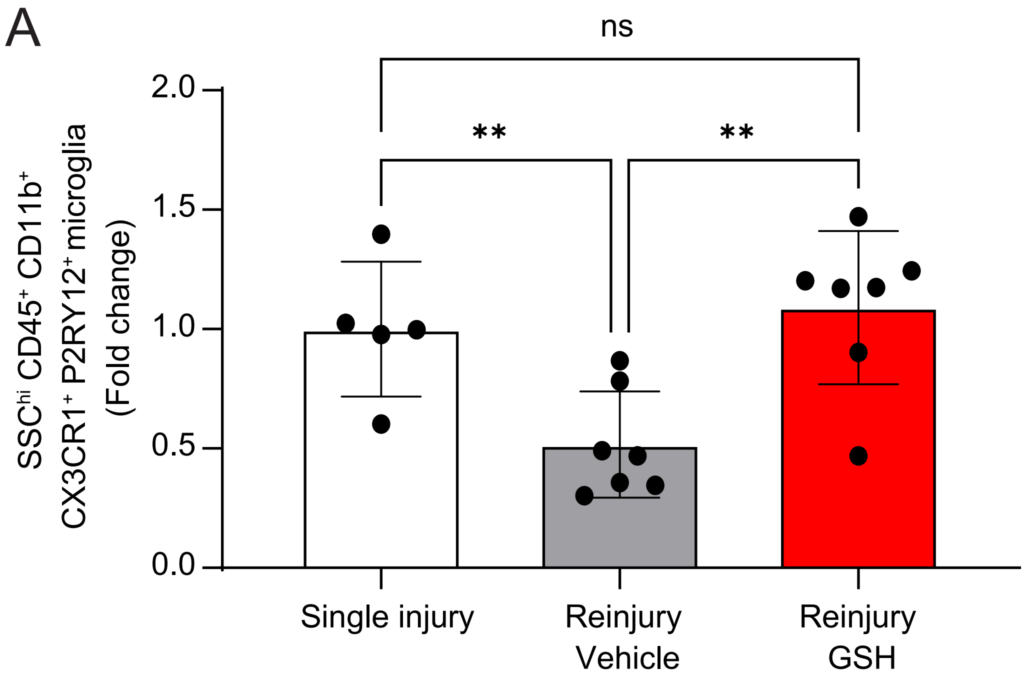
A
ns
**
**
2.0
1.5
1.0
0.5
0.0
SSC^{hi} CD45^{+} CD11b^{+}
CX3CR1^{+} P2RY12^{+} microglia
(Fold change)
Single injury
Reinjury
Vehicle
Reinjury
GSH

Figure S7

SUPPLEMENTARY FIGURE LEGENDS

Supplemental Figure 1. Reinjury promotes death of multiple parenchymal cell types

(a,c,e,g,i,k) Representative *xy* maximal projection *z*-stacks (20μm in depth) of reinjured neocortex versus the uninjured contralateral hemisphere stained with DAPI (nucleated cells), PI (dead cells), anti-NeuN (neurons), anti-Iba1 (myeloid cells), anti-APC (oligodendrocytes), or anti-CD31 (endothelial cells). **(a,c,e)** DAPI (blue), PI (red), NeuN (green), and Iba1 (white) were stained and analyzed together. **(i)** APC and **(k)** iv CD31 were also visualized in combination with DAPI and PI (not shown) to facilitate identification of the lesion. **(g)** ALDH1 images (red) depicts TdTomato signal detected in *Aldh1*$^{CreER/+}$ Stop$^{fl/fl}$ TdTomato mice. Scale bar: 20μm **(b)** Quantification of cellularity by selecting only propidium iodide (PI) negative, 4′,6-diamidino-2-phenylindole (DAPI) positive cells. (d,f,h,j) Quantification of cell density by selecting spots positive for NeuN, Iba1, ALDH1, and APC, respectively. (l) Quantification of percent CD31 volume per total tissue volume. Each symbol (b,d,f,h,j,l) depicts an individual mouse. Bar graphs represent two-independent pooled experiments with 3-5 mice per group per experiment. Data were normalized to average uninjured contralateral hemispheres per experimental day and displayed as the mean fold change ± S.D. with ns (not significant) ≥0.5, **P≤0.01, and ****P≤0.0001 (two-tailed Student’s t-test).

Supplemental Figure 2. Gating strategy for resident myeloid cells

(a) Representative FACS plots from a naïve mouse show the gating strategy used to identify resident myeloid cells. Single cells were initially gated as viable (Live/Dead^{-}) leukocytes (CD45^{+}). Cells were then gated CD19^{-} TCRβ^{-} Ly6C^{-} and Ly6G^{-} to exclude B cells, T cells, inflammatory monocytes and neutrophils. Resident myeloid cells were then identified as being CX3CR1^{+}. **(b)** The bar graph shows the absolute number of each microglia subset per experimental group. Each symbol depicts an individual mouse. Bar graphs are representative of 2 independent experiments with 4-5 mice per experimental group. Data are displayed as mean ± S.D. with **P*≤0.05, ***P*≤0.01, and ****P*≤0.001 (multiple *t*-tests with Holm-Sidak multiple comparisons test).

Supplemental Figure 3. Injury generates diverse microglia subsets (a) Quantification of relative size (FSA-A) and granularity (SSC-A) of each microglia subpopulation per experimental group. **(b)** Quantification of geometric mean fluorescence intensity (GFMI) of CD11b, CX3CR1, F480, P2RY12, and CD206 expression on each microglia subpopulation per experimental group. Each symbol (a,b) depicts an individual mouse. Bar graphs are representative of 2 independent experiments with 4-5 mice per experimental group. Data are displayed as mean ± S.D. with ns (not significant) ≥0.5, *P≤0.05, **P≤0.01, and ***P≤0.001 (multiple t-tests with Holm-Sidak multiple comparisons test).

Supplemental Figure 4. Resident myeloid cells support the glia limitans superficialis following single injury not reinjury

(a) Representative *xz* maximally projected *z*-stacks (300μm in depth) of SR101 leakage (white) applied transcranially through the skull (green) in single injury mice fed either control or CSF-1R inhibitor (PLX3397) chow. Glia limitans depicted as red-dashed line. Scale bar: 20μm. **(b)** Quantification of SR101 leakage by MFI. **(c)** Representative *xz* maximal projections *z*-stack (300μm in depth) of SR101 leakage (white) applied transcranially through skull (green) in reinjury mice fed either control or CSF-1R inhibitor chow. Glia limitans depicted as red-dashed line. Scale bar: 20μm. **(d)** Quantification of SR101 leakage by MFI. **(b,d)** Bar graphs represent two-independent, pooled experiments with 4-7 mice per group per experiment. Data were normalized to control chow mouse per experimental day and displayed as the mean fold change ±S.D. with ns (not significant) ≥0.5 and $^*P \leq 0.05$ (two-tailed Student's *t*-test).

Supplemental Figure 5. Reactive oxygen species are spread diffusely throughout the meninges

Representative xy (50μm in depth), yz (150μm in depth), xz (150μm in depth) maximal projections captured by two-photon microscopy show the distribution of reactive oxygen species (ROS; white) stained transcranially with AmplexTM Red Reagent (white) in a LysMgfp/+ mouse immediately following reinjury at 24 hrs. The diffuse ROS staining localized primarily to the meninges in regions with and without LysMgfp/+ myelomonocytic cells (green). Scale bar: 50μm. Image represents two-independent experiments with 1-3 mice per experiment.

Supplemental Figure 6. Glutathione administration preserves surface associated astrocytes

(a) Representative *xy* maximal projections *z*-stack (40µm in depth) show ALDH1+ astrocytes (green) in reinjured *Aldh1*$^{CreER/+}$ Stop$^{fl/fl}$ TdTomato mice following treatment with vehicle (PBS) or GSH. Vasculature is denoted with dotted white lines. All injury mice were treated immediately following reinjury and sacrificed at 30hr post-initial mTBI. Images are representative of 3-5 independent mice per group from two-independent experiments. Scale bar: 40µm. **(b)** Bar graph represents two-independent, pooled experiments with 3-5 mice per group per experiment. Data were normalized to control PBS-treated mice per experimental day and displayed as the mean fold change ± S.D. with ****$P \leq 0.0001$ (two-tailed Student's *t*-test).

Supplemental Figure 7. Glutathione administration preserves microglia (a) Quantification of protective microglia phenotype. Single cells were initially gated as leukocytes ($CD45^+$). Cells were then gated as $CD19^-$ $TCR\beta^-$ $Ly6C^-$ and $Ly6G^-$ to exclude B cells, T cells, inflammatory monocytes, and neutrophils, respectively. Neuroprotective microglia were identified as being SSChi CX3CR1+ CD11b+ P2RY12+. Bar graph represents two-independent, pooled experiments with 2-5 mice per group per experiment. Data were normalized to control single injury mice per experimental day and displayed as the mean fold change ± S.D. with **$P \leq 0.01$ (two-tailed Student's t-test).

SUPPLEMENTARY VIDEO LEGENDS

Supplemental Video 1. Microglia dynamics in naïve and mTBI mice.

Representative *xy* (top) and *xz* (bottom) maximally projected time lapses show $CX3CR1^{gfp/+}$ myeloid cells (green) and vasculature labeled with Evans blue (red) captured by two-photon microscopy in naïve (Part 1), single injury 6 hours (Part 2), single injury 30 hours (Part 3), and reinjury 30 hours (Part 4) mice. In Part 2 $CX3CR1^{gfp/+}$ microglia can be observed forming honeycomb-like structures along the glia limitans superficialis. At 30 hours following a single injury, glia limitans microglia are mostly amoeboid (Part 3). Many of these cells die following reinjury at 24 hours. The regions outlined with white dotted lines in Part 4 show GFP leaking out of dying $CX3CR1^{gfp/+}$ cells. Images are representative of 4 independent mice per group.

Supplemental Video 2. Myelomonocytic influx after repeat mTBI depends on LFA-1 / VLA-4.

Representative *xy* (top) and *xz* or *yz* (bottom) maximally projected time lapses show $LysM^{gfp/+}$ myelomonocytic cells (green) and vasculature labeled with Evans blue (red) captured at 30 hours by two-photon microscopy in single injury (Part 1), control IgG treated reinjury (Part 2), and αLFA-1/VLA-4 treated reinjury (Part 3) mice. Thirty hours following a single mTBI, LysMgfp/+ myelomonocytic cells are found almost exclusively in the meninges above the glia limitans superficialis (Part 1). Reinjury massively increases influx of these cells – some of which enter the parenchyma (Part 2). Administration of αLFA-1/VLA-4 blocks most of this recruitment (Part 3). Images are representative of 8 independent experiments with 2-4 mice.

Supplemental Video 3. Glutathione treatment reduces influx of myelomonocytic cells after reinjury.

Representative *xy* (top) and *xz* (bottom) maximally projected time lapses show LysM$^{gfp/+}$ myelomonocytic cells (green) and vasculature labeled with Evans blue (red) captured at 30 hours by two-photon microscopy in vehicle (Part 1) or glutathione (GSH, Part 2) reinjury mice. Images are representative of three independent experiments with 1-3 mice per group.
